# Microarchitecture, but Not Bone Mechanical Properties, Is Rescued with Growth Hormone Treatment in a Mouse Model of Growth Hormone Deficiency

**DOI:** 10.1155/2012/294965

**Published:** 2012-03-13

**Authors:** Erika Kristensen, Benedikt Hallgrímsson, Douglas W. Morck, Steven K. Boyd

**Affiliations:** ^1^Department of Mechanical and Manufacturing Engineering, Schulich School of Engineering, University of Calgary, 2500 University Dr NW, Calgary, AB, Canada T2N 1N4; ^2^McCaig Institute for Bone and Joint Health, University of Calgary, Calgary, AB, Canada T2N 4N1; ^3^Department of Cell Biology and Anatomy, Faculty of Medicine, University of Calgary, Calgary, AB, Canada T2N 4N1; ^4^Department of Biological Sciences, Faculty of Science, University of Calgary, Calgary, AB, Canada T2N 4N1; ^5^Department of Comparative Biology and Experimental Medicine, Faculty of Veterinary Medicine, University of Calgary, Calgary, AB, Canada T2N 4N1

## Abstract

Growth hormone (GH) deficiency is related to an increased fracture risk although it is not clear if this is due to compromised bone quality or a small bone size. We investigated the relationship between bone macrostructure, microarchitecture and mechanical properties in a GH-deficient (GHD) mouse model undergoing GH treatment commencing at an early (prepubertal) or late (postpubertal) time point. Microcomputed tomography images of the femur and L4 vertebra were obtained to quantify macrostructure and vertebral trabecular microarchitecture, and mechanical properties were determined using finite element analyses. In the GHD animals, bone macrostructure was 25 to 43% smaller as compared to the GH-sufficient (GHS) controls (*P* < 0.001). GHD animals had 20% and 19% reductions in bone volume ratio (BV/TV) and trabecular thickness (Tb.Th), respectively. Whole bone mechanical properties of the GHD mice were lower at the femur and vertebra (67% and 45% resp.) than the GHS controls (*P* < 0.001). Both early and late GH treatment partially recovered the bone macrostructure (15 to 32 % smaller than GHS controls) and the whole bone mechanical properties (24 to 43% larger than GHD animals) although there remained a sustained 27–52% net deficit compared to normal mice (*P* < 0.05). Importantly, early treatment with GH led to a recovery of BV/TV and Tb.Th with a concomitant improvement of trabecular mechanical properties. Therefore, the results suggest that GH treatment should start early, and that measurements of microarchitecture should be considered in the management of GHD.

## 1. Introduction

Growth hormone (GH) plays an important role in the growth of bone, and a lack of GH during development results in a delayed bone age and reduced bone mineral density [1]. A GH deficiency in childhood is believed to cause an increased fracture risk and risk of osteoporosis later in life with GH treatment potentially mitigating this risk [2, 3]. However, the evidence to support this is controversial, and it has been suggested that GH deficiency should no longer be listed as a cause of osteoporosis in children [4].

A high prevalence of fractures has been observed in adults with hypopituitarism including GH deficiency with onset in childhood or adulthood [5–7], and these increased fracture rates have been mainly attributed to a lack of GH [5, 6]. Children, adolescents, and adults with childhood-onset isolated GH deficiency had an increased fracture risk [6, 8], while a separate study found a low prevalence of fractures in this same population [7]. GH treatment has been shown to have a protective effect by reducing fracture frequency although the mechanisms behind this are not clear [8, 9].

Studies have demonstrated that bone mineral density is reduced with GH deficiency and that GH treatment normalizes BMD [10–12]. These studies have relied primarily on two-dimensional measures of density, which are affected by bone size [13]. When size corrections or volumetric density measurements are used, most studies suggest that a GH deficiency results in normal or near-normal BMD [12, 14, 15], while some report below normal values [16].

Parameters that quantify trabecular microarchitecture have been shown to relate strongly to bone strength [17]. Transiliac bone biopsies reveal no histomorphometric differences between the trabecular bone of GH-deficient men and controls [18], and GH treatment in this same population showed no changes in trabecular structure [19, 20]. In moderately GH-deficient rats, trabecular microarchitecture was significantly compromised with a smaller number of thinner trabeculae and a reduced connectivity density [21]. In a previous study assessing changes in trabecular microarchitecture during growth using *in vivo* microcomputed tomography (*μ*CT), we showed that GH-deficient mice have compromised vertebral trabecular microarchitecture. Treatment commencing prepuberty rescued the quantity of trabecular bone but not the structure, while treatment after puberty had no detectable effect. GH-deficient mice had an increased number of thin trabeculae compared to GH-sufficient mice, and GH treatment resulted in a trend towards further increases in the number of trabeculae [22]. To expand on this work, in this study, we sought to determine the effects of this altered microarchitecture on bone mechanical properties as well as incorporate measures of bone macrostructure using high-resolution *in vitro* *μ*CT.

Some investigators have studied the effects of GH on bone strength. Growth hormone was shown to increase bone strength in both young and old GH-sufficient rats, and this increase has been attributed almost solely to a larger bone size [23, 24]. In a rat model of osteoporosis, GH treatment was shown to reverse the loss of bone strength by increasing the ultimate stress and Young's modulus to normal levels [25]. GH-deficient mice and rats have impaired cortical bone strength which was attributed to a reduction in cortical diameter, and in mice the loss of strength was proportional to the degree of GH deficiency [21, 26].

It is unclear what role microarchitecture plays in bone strength with GH deficiency, particularly since previous work indicates that increased fracture risk may simply be due to a smaller bone size. The purpose of this study is to understand the role of trabecular and cortical bone mechanical properties and their relation to microarchitecture and macrostructure in a GH-deficient mouse model. Our previous *in vivo* study focused on temporal changes in microarchitecture with GH treatment at the fourth lumbar vertebra. Here we use high-resolution *in vitro* *μ*CT imaging at multiple skeletal sites to address the hypothesis that GH-deficient mice have compromised trabecular microarchitecture in addition to small bone size that together lead to a reduction in mechanical properties. We test the hypothesis that commencement of GH treatment before puberty results in a recovery of mechanical properties that accompany the microarchitectural changes in contrast to when treatment is started after puberty.

## 2. Methods

### 2.1. Experimental Design

Ghrhr homozygous “little” (*lit/lit*) mice with a C57Bl/6 background have impaired GH synthesis and release although injection of exogenous GH can stimulate growth [27]. Heterozygous (*lit/+*) mice have normal GH synthesis and release and are comparable to wild-type C57Bl/6 mice. Breeding pairs of homozygous males and heterozygous females were purchased from The Jackson Laboratory (Bar Harbour, Maine, USA). This breeding arrangement was used as female homozygous mice have impaired first lactations and, therefore, may lose their first litter [28]. Offspring were weaned at 21 days old and assigned to one of five experimental groups: GH-sufficient (*lit/+*) controls, GH-deficient (*lit/lit*) controls, early treatment (*lit/lit*), late treatment (*lit/lit*), and saline injection control (*lit/lit*). A limitation of this mouse model was the large amount of time required to breed the animals in-house (>2 years), resulting in the necessity of using both sexes to obtain a sufficient sample size for the experiment. All experimental groups consisted of five male and five female mice, with the exception of the saline injection control group, which was composed of three male and three female mice. All animals were housed in groups of the same sex and allowed *ad libitum* access to standard rodent chow and water.

Early treatment and late treatment groups received daily subcutaneous injections of 25 *μ*g of mouse recombinant growth hormone (Dr. A. F. Parlow, National Hormone and Peptide Program). The use of daily injections attempts to mimic the physiological release of GH, which occurs in a pulsatile manner with the largest peak release of GH occurring during sleep [29]. The dose (25 *μ*g per mouse per day) was chosen as it is within the wide range of doses which stimulated growth in mice and rats [23, 24, 30]. Although recombinant human GH is often used to treat GH deficiency in animal experiments, antibody production can limit the efficacy of this treatment [31].

Growth hormone was prepared as detailed by Kristensen et al. [22]. The early treatment group received daily subcutaneous (sc) injections of 25 *μ*g of GH from age 21 days to 60 days, while the late treatment group received sc injections of 25 *μ*g of GH from age 35 days to 60 days. At the age of 21 days, mice have not yet started puberty, while the age of 35 days corresponds approximately to the end of puberty [32]. Daily sc injections were used instead of continuous dosing with infusion pumps due to the small size of the mice at the study onset. The GH-deficient mice remained below the minimum body weight recommended for pump implantation until the day 45 time point. The saline injection control mice received daily sc injections of saline from age 21 days to 60 days to determine if the stress of daily injections has an effect on bone microarchitecture or bone mechanical properties. All procedures were reviewed and approved by the University of Calgary Health Sciences Animal Care Committee, and experiments were conducted in compliance with this approval. Animals were cared for as per the guidelines of the Canadian Council on Animal Care.

### 2.2. Imaging

At 60 days of age, the mice were euthanized by CO_2_ inhalation, and the fourth lumbar vertebra (L4) and left femur were dissected free and all soft tissue removed. Tissue was stored at −30°C in saline-soaked gauze until ready for use. High-resolution *μ*CT images (*μ*CT35, Scanco Medical AG) of the entire L4 vertebra and femur were obtained with an integration time of 800 ms, and X-ray settings of 55 kVp, and 145 *μ*A. The L4 vertebrae were scanned resulting in an isotropic voxel size of 3.5 *μ*m, and the data were subsequently binned to 14 *μ*m (Image Processing Language (IPL) v5.08b, Scanco Medical AG). The femora were scanned with an isotropic voxel size of 10 *μ*m. All images were Gaussian filtered (vertebra: sigma = 1.0, support = 1; femur: sigma = 1.2, support = 2), and a fixed threshold of 22.0% of the maximal gray scale value was applied to extract the mineralized tissue. The bones were positioned so that the long axes of the vertebral body and the femur were aligned with the *z* axis (IPL v5.08b, Scanco Medical AG).

### 2.3. Bone Geometry and Trabecular Microarchitecture

#### 2.3.1. Vertebra

 To calculate the vertebral body height, the number of slices containing vertebral body bone was counted and multiplied by the slice thickness. The cross-sectional area (CSA) was obtained by calculating the area within a hand-drawn contour of the vertebral body on the slice located midway through the body. To quantify the trabecular microarchitecture, the trabecular region of the vertebral body was extracted by hand-drawing contours on every fourth slice in the trabecular region between the growth plates and applying a semiautomated morphing algorithm to the intervening slices. Bone microarchitectural parameters were calculated: bone volume ratio (BV/TV), trabecular number (Tb.N), trabecular thickness (Tb.Th), trabecular separation (Tb.Sp), structure model index (SMI), and connectivity density (Conn.D) (IPL, v5.08b, Scanco Medical AG). The trabecular bone tissue mineral density (TMD) was calculated by normalizing the mineral content (as determined by X-ray attenuation) by the trabecular bone volume.

#### 2.3.2. Femoral Diaphysis

To calculate the full femur length, the number of slices containing bone was summed and multiplied by the slice thickness. All femur analyses (except full femur length) were performed at femur diaphysis only, which is a region consisting primarily of cortical bone. This is in contrast to the vertebra which is mainly composed of trabecular bone. These two regions were selected so that both cortical bone and trabecular bone properties could be assessed.

The remainder of the femur analyses required that a section of the femoral shaft be extracted. In order to analyse comparable regions of each bone, the shaft length was chosen based on the sample's aspect ratio. Therefore, the length of the shaft (*L*) was unique for each model and was set based on an aspect ratio of 6.0 multiplied by the shaft diameter. The shaft diameter was calculated for a section of the femoral shaft with a length equal to half of the entire bone length. Thus, the final femoral shaft models were created by extracting a section of the shaft of length *L*, centered about the shaft midpoint which was defined as 53% of the femur length as measured from the proximal end [33]. The average cortical thickness (Ct.Th) of the femoral shaft model was calculated by direct methods [27] (IPL, v5.08b, Scanco Medical AG).

### 2.4. Finite Element Analysis

The finite element (FE) method is a computational approach used to model the mechanical behavior of materials and can determine mechanical properties of bone [34, 35]. For this study, *μ*CT images are used as the basis of the model, as each voxel in the image is converted into an element in the mesh using the voxel conversion approach [36, 37].

#### 2.4.1. Vertebra

Two FE analyses were performed on each vertebra: a uniaxial compression of the whole bone, as well as the calculation of the full stiffness matrix for a subvolume of vertebral body trabecular bone [34].

**Vertebra Whole Bone**

**Figure 1:**
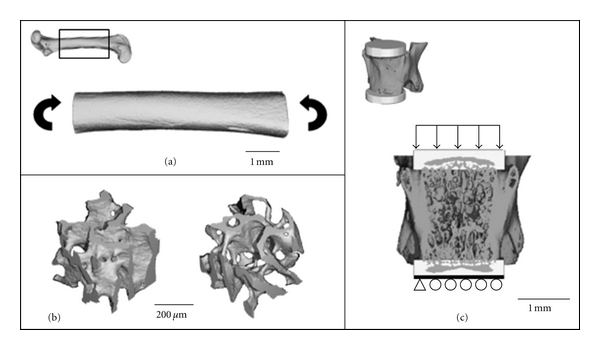
Finite element models. (a) Femur, positioned with condyles down, loaded in pure bending to an angle of 0.01 radians. (b) Cubes of trabecular bone extracted from the distal region of the L4 vertebral body. On the left, bone from the GH-sufficient mouse and, on the right, bone from a GH-deficient mouse are pictured, each with the median apparent modulus in their respective groups. (c) Vertebrae with endcaps digitally encasing the vertebral body endplates. Vertebrae were loaded in compression to 1% strain.

Homogeneous, linear finite element models of the extracted mineralized phase of the vertically aligned whole vertebra images were created. Plano-parallel loading surfaces were generated by digitally inserting cylindrical endcaps onto the vertebral endplates (Figure 1(c)). The diameter of the endcaps was selected to enclose the entire endplate. The top endcaps were assigned a thickness of 0.42 mm, while the bottom endcaps were 0.35 mm thick. Vertebral processes were trimmed so that they did not extend beyond the vertebral endplates. The voxel conversion approach was used to transform each voxel in the image into a hexahedral element in the FE model. A Young's modulus of 19 GPa and a Poisson's ratio of 0.3 were assigned to bone [38], while the endcaps were assigned material properties equivalent to bone cement (PMMA): Young's modulus = 2.5 GPa, Poisson's ratio = 0.3 [39]. The nodes on the bottom surface of the lower endcap were constrained in the *Z* direction but free to move in the *X* and *Y* directions. The nodes on the top surface of the upper endcap were free to move in the *X* and *Y* directions and a uniaxial displacement (*δ*) equivalent to 1% strain was applied in the *Z* direction.

The models were solved using custom FE software (FAIM v4.0) on a desktop computer (Mac, OS X, Version 10.5.6; 2 × 2.8 GHz Quad-Core Intel Xeon). The meshes generated from the whole vertebra models contained an average of 2.6 million nodes and 2.1 million elements. The output of the models was the reaction force (*F*) required to induce 1% strain. The axial rigidity (*AE*) was calculated as

(1) $$AE=\frac{Fh}{\delta },$$

where *A* is the cross-sectional area of the vertebral body, *E* is the apparent level Young's modulus, and *h* is the vertebral body height.

**Vertebra Trabecular Subvolume**

A cubic subvolume of trabecular bone (130 × 130 × 130 voxels) was extracted from the images with an isotropic voxel size of 3.5 *μ*m (Figure 1(b)), and mineralized bone was identified as described previously. The voxel conversion approach was applied, and three uniaxial strain compression and three uniaxial shear strain tests were performed. The models were solved using IPL (v5.11/FE-V01.15, Scanco Medical AG). The meshes generated from the trabecular subvolume images contained an average of 572,335 nodes and 471,255 elements. The stiffness matrix in the orthotropic principal coordinate system was calculated [34], and the apparent Young's modulus in the first principal direction was obtained.

#### 2.4.2. Femoral Diaphysis

A homogeneous, linear finite element analysis was performed to determine the flexural rigidity of the femoral diaphysis. As the finite element results are calculated with the use of beam theory, and because mechanical properties calculated from beam theory are highly dependent on the sample's aspect ratio [33], this parameter was held constant for all models. Therefore, the shaft length was selected for each model based on an aspect ratio of 6.0, as described previously (Section 2.3.2). The voxel conversion approach was used to convert the femoral diaphysis image into a FE model. A Young's modulus of 19 GPa and a Poisson's ratio of 0.3 were assigned to all bone elements [38]. The models were loaded in pure bending to an angle of 0.01 radians, and this load was applied with the femoral condyles facing downwards, which is a position consistent with that used for mechanical testing (Figure 1(a)). The models were solved using custom FE software (FAIM v4.0) on a desktop computer (Mac, OS X, Version 10.5.6; 2 × 2.8 GHz Quad-Core Intel Xeon). The meshes generated for finite element analysis resulted in an average of 5.3 million nodes and 4.8 million elements for the full femur models. The flexural rigidity (*EI*) was calculated based on the moment (*M*) required to bend the model to an angle of 0.01 radians (*θ*) and the length of the femoral shaft (*L*)

(2) $$EI\;=\frac{ML}{\theta }.$$

### 2.5. Statistics

Statistical significance of the parameters was tested by an analysis of variance (ANOVA). Where between-subjects significance was observed, a post hoc Tukey's test was used to determine differences between group means. All statistical tests were performed with PASW Statistics version 17.0 (SPSS Inc, Chicago, USA). Differences were considered statistically significant with *P* < 0.05 for all tests.

## 3. Results

There were no significant differences between the GH-deficient and saline injection control mice for any parameter, supporting the assumption that any stress due to daily injections did not have a detectable effect on bone size, microarchitecture, or mechanical properties.

### 3.1. TMD and Bone Macrostructure

**Figure 2:**
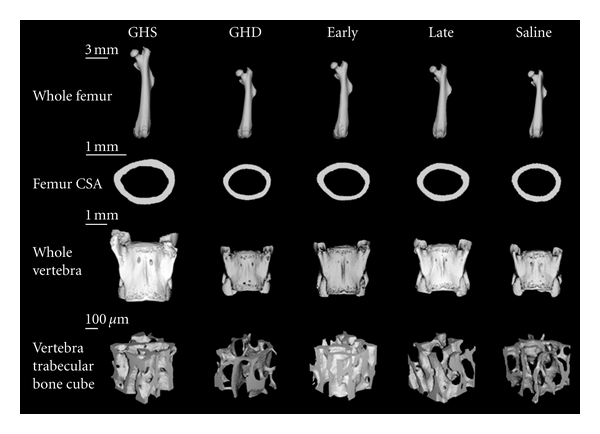
Bone macrostructural results. From top to bottom, the rows display the whole femur, the femur cross-section (CSA), the whole vertebra, and cubes of vertebral trabecular bone. For the whole femur, the animal with the median femoral length in each group is displayed, and this same animal's femur cross-section (at the shaft midpoint) is shown in the second row. For the whole vertebra, the mouse with the median vertebral body height in each group is shown, and the animal with the median BV/TV in each group is depicted in the bottom row. GH sufficient (GHS), GH deficient (GHD), early treatment (early; daily GH injections from age 21 to 60 days), late treatment (late; daily GH injections from age 35 to 60 days), and saline control (Saline; daily saline injections from age 21 to 60 days).

 All tissue mineral density (TMD) and bone macrostructure results are listed in Table 1, and the macrostructure results are displayed in Figure 2. There were no detected differences in TMD between any of the groups. The GH-deficient mice had a significantly smaller vertebral body height, vertebral body CSA, femoral length, femoral cortical thickness, and femoral CSA than the GH-sufficient mice (25% to 43% smaller than GHS, *P* < 0.001). Growth hormone treatment was associated with increases in these dimensions so that they were larger than the GH-deficient mice regardless of the age of onset of treatment (10% to 23% larger than GHD, *P* < 0.003). However, all of these morphological parameters remained smaller in both treatment groups compared to the GH-sufficient mice (15% to 32% smaller than GHS, *P* < 0.001), and thus GH treatment only resulted in the partial rescue of bone macrostructure. The only morphological parameter that showed an effect of early treatment was femur length, where a significantly longer bone length was observed with early GH treatment as compared to late treatment (3% longer than Late, *P* = 0.041). The femoral medullary CSA was significantly smaller in the GH-deficient, early treatment, and late treatment mice as compared to the GH-sufficient mice (29 to 39% smaller than GHS, *P* < 0.001). There was a trend towards an increase in medullary CSA with GH treatment that was significant for the late treatment group only (11 to 16% larger than GHD, *P* = 0.023).

### 3.2. Vertebral Microarchitecture

All vertebral microarchitecture results are displayed in Table 2, and a cube of vertebral trabecular bone is displayed for the animal with the median BV/TV in each group in Figure 2. The trabecular bone volume ratio was significantly smaller in the GH-deficient L4 vertebrae as compared to the GH-sufficient vertebrae (20% smaller than GHS, *P* = 0.031). Growth hormone treatment was associated with an increase in the bone volume ratio (BV/TV) in both the early and late treatment mice (14 to 25% larger than GHD); however, this increase was significant only between the early treatment and GH-deficient mice (*P* = 0.039), with no significant differences between the early treatment and GH-sufficient mice. There were no significant differences in BV/TV between the late treatment and GH-sufficient or GH-deficient mice, indicating a partial recovery. These same trends were observed for SMI, with a more rod-like structure in the GH-deficient mice compared to the GH sufficient mice (68% larger than GHS, *P* = 0.002). A recovery of SMI was observed in the early treatment group with a lack of a significant difference from the GH-sufficient mice but a significantly smaller SMI than the GH-deficient mice (32% smaller than GHD, *P* = 0.021). Again, a partial recovery was evident in the late treatment group as no significant differences existed between this group and the GH-sufficient or GH deficient groups.

The GH-deficient, early treatment, and late treatment groups had significantly thinner trabeculae than the GH sufficient groups (8 to 19% smaller than GHS, *P* < 0.05). Early treatment led to some recovery of Tb.Th as this parameter was significantly larger in the early treatment mice than the GH-deficient mice (14% larger than GHD, *P* = 0.001).

For Tb.N, Tb.Sp, and Conn.D, there were no detected differences among any of the groups.

### 3.3. Mechanical Properties

The results for the mechanical properties are displayed in Table 3. The GH-deficient, early treatment, and late treatment animals all have significantly less vertebral axial rigidity than the GH-sufficient animals (27 to 45% smaller than GHS, *P* < 0.001). However, GH treatment resulted in partial recovery of this parameter as both the early and late treatment mice have a larger axial rigidity than the GH-deficient mice (24 to 33% larger than GHD, *P* < 0.05).

Similar to the vertebrae, the femoral diaphyseal flexural rigidity was significantly greater in the GH-sufficient group as compared to the GH-deficient, early treatment, and late treatment groups (52 to 67% smaller than GHS, *P* < 0.001). Both early and late treatment resulted in partial recovery (42 to 43% larger than GHD, *P* < 0.05).

The apparent Young's modulus, reflecting the vertebral trabecular bone mechanical properties, was significantly smaller in the GH-deficient as compared to the GH-sufficient mice (37% smaller than GHS, *P* = 0.012). There was a recovery of this parameter with early growth hormone treatment leading to a significant difference from GH-deficient mice (56% larger than GHD, *P* = 0.02) and no detected difference from GH-sufficient mice—the trabecular bone mechanical properties appear to have been rescued. Late treatment may have also resulted in partial recovery of the trabecular bone mechanical properties, but not as clearly as for early treatment due to the lack of detected differences from either GH-deficient or GH-sufficient groups.

## 4. Discussion

This study provided novel insight into the relationships between cortical and trabecular bone macrostructure, microarchitecture, and mechanics in a GH-deficient model. It demonstrated that GH deficiency results in a deterioration of bone size, microarchitecture, and mechanical properties, and that GH treatment partially restored the bone size, regardless of when the treatment was started (early or late). Early treatment fully restored some important aspects of the vertebral trabecular microarchitecture, while partial recovery was observed in other aspects. In addition, late treatment resulted in a less complete recovery of the trabecular microarchitecture. The macrostructure and microarchitectural changes were related to bone mechanical properties at two skeletal sites (L4 vertebra and femoral diaphysis). It was found that although both early and late GH treatment resulted in partial recovery of the mechanical properties, the remaining deficit could be explained by compromised macrostructure and not microarchitecture because it was shown that, at least in the early treatment group, trabecular mechanical properties were fully recovered.

Others have shown that GH deficiency results in a small bone size, and this is consistent with the results observed in this study. The GH treatment did not result in the recovery of normal bone size, and similar trends were observed in human studies where GH-deficient children were significantly shorter than normal after six years of treatment [10]. As response to GH treatment in GHD children and animals depends on GH dose [24, 40], it is possible that the dose given to the mice in this study was not large enough to result in full catch-up growth. While this dose is consistent with doses that were shown to induce bone and muscle growth in both GH-sufficient and -deficient rodents [23, 24, 30], most of those studies used a dose scaled to body weight. Thus as the animals grew and increased body mass, the amount of GH administered was increased. Additionally, it has been shown that, using a fixed dose, the growth response blunts with time [41]. In this study, a constant dose that was independent of body mass was administered, which could limit the response to GH treatment.

Since the postnatal period (1 to 23 days of age) is the most rapid growth phase for long bones in the mouse [42], it is likely that GH treatment would be more effective in restoring normal bone length if administered during this phase. However, there are practical challenges associated with treating mice before the age of weaning at 21 days. The small size of the pups prevents infusion pump implantation, and potential rejection of the pup by its mother prohibits daily manual injection. Thus, it was not possible to treat these mice before weaning. However, continuing treatment past the day 60 endpoint may also have resulted in a more complete recovery of bone dimensions, as the duration of GH treatment can have an impact on the response to GH therapy [11].

The macrostructural results for the femur suggest that the early treatment mice are at a more mature stage of bone development than the late treatment mice at the study endpoint. Normal bone growth in C57Bl/6 mice involves rapid periosteal expansion until 28 days of age, when expansion slows [43]. Endosteal expansion reaches its peak at 56 days of age and afterwards decreases, showing a net resorption of endosteal bone before 56 days and a net apposition after this point [43]. The medullary CSA was larger in the late treatment group and there was a trend towards increased cortical thickness in the early treatment group. This indicates that the early treatment group was likely in the period of endosteal apposition while the late treatment group was still in the endosteal resorption phase at 60 days of age, suggesting that the early treatment group is at a more advanced stage of cortical bone development.

GH-deficient mice exhibited a significant deterioration of their trabecular microarchitecture, while mice treated before puberty exhibited recovery of many aspects. This is consistent with our previous longitudinal study of these same mice that was based on lower resolution *in vivo* imaging. In that study, we found a significantly larger number of trabeculae in GH-deficient, early treatment, and late treatment animals as compared to GH-sufficient animals, with a trend towards further increases with GH treatment [22]. Although not significant, the data presented here indicates that there is a trend towards an increased number of trabeculae in all of the GH-deficient groups, with more prominent increases in the treatment groups. Combined with the increase in trabecular thickness with treatment, these results suggest that mice receiving GH treatment have normalized bone volume ratio through both increased number and thickness of trabeculae.

An interesting result is that while bone size was not restored in the GH-treated mice, some aspects of the microarchitecture were indeed rescued. Thus, decreases of whole bone mechanical properties can be attributed to smaller bones, but not to deficits in the bone microarchitecture. If this animal model reflects the clinical situation, these results suggest that assessments of the effectiveness of GH therapy must go beyond measuring bone size and density and should include the effect of GH on microarchitectural changes. With emerging technologies (e.g., high-resolution peripheral quantitative computed tomography), this approach to clinical assessment may become feasible in the near future.

There were no differences in tissue mineral density detected between any of the groups, and this is in agreement with the majority of human studies measuring volumetric or size-adjusted BMD but differs from GHD animal models where decreased BMC and BMD were observed [44], including our recent *in vivo* study [22]. It is probable that the discrepancy is due to differences in image resolution which influence partial volume effects and TMD estimates. Considering the limitations of TMD measurements with the polychromatic X-ray source used in *μ*CT, it may be necessary in the future to use synchrotron *μ*CT. Regardless, if there is truly an effect of GH treatment on TMD, the effect is likely small. The rationale for including the assessment of TMD in this study was that it confirms that the use of a homogeneous tissue modulus in the finite element analysis is a reasonable approach.

A lack of growth hormone led to a reduction of femoral flexural rigidity and vertebral axial rigidity, and partial recovery of both of these parameters was evident with GH treatment. These parameters are dependent on bone size and therefore are affected by the reduced bone size in GH-deficient animals and partial size recovery with GH therapy. Since the structure of trabecular bone plays a role in mechanical behavior, the mechanics results for the vertebrae are not simply a function of size, but of the structure of the trabecular bone as well. The FE results for isolated trabecular bone subvolumes indicated that the trabecular structure of GH-deficient animals was significantly weaker, and that early GH treatment restored the trabecular mechanical properties. Therefore, this further supports the notion that the deficits in vertebral axial rigidity in early treatment animals are likely caused by a smaller bone size, and not by a weakened microarchitecture. Although early treatment does not fully restore bone size, resulting in a net deficit in the whole bone mechanical properties, the recovery of trabecular mechanical properties is likely valuable for reducing fracture risk in the long term. In the late treatment mice, a similar pattern was observed; however, the trabecular mechanical properties were not completely rescued. Together these results confirm support for the previous finding that it is important for GH treatment to be commenced early.

There were limitations in this study that deserve mention. First, the finite element analyses did not incorporate variations in tissue modulus and therefore only assessed the contributions of size and structure on mechanical properties. Nevertheless, it would have been difficult to justify incorporating tissue mineral density due to the lack of significant differences found among groups. Second, in the small mouse bones, it was challenging to extract trabecular subvolumes with at least five trabeculae as suggested by Harrigan et al. [45] for continuum assumptions. This was due to the small size and irregular shape of the vertebral bodies in the mouse and may be justification to consider a larger animal model in the future (e.g., rat). Finally, it should be noted that the findings in this study using a mouse model are not directly applicable to human patients, and further research is needed to explore the importance of timing of GH treatment on bone health in people. This research would be particularly interesting to pursue using new clinical 3D technologies such as high-resolution peripheral quantitative computed tomography on patient cohorts to assess human bone microarchitecture (rather than only bone mineral density by DXA).

In summary, GH deficiency resulted in reduced bone macrostructure, a deteriorated trabecular microarchitecture, and decreased bone mechanical properties as compared to GH-sufficient controls. Treatment with sc GH was unable to fully recover bone size although the trabecular mechanical properties in the early treatment group were fully restored despite the net reduction in whole bone strength. Therefore, the benefits of GH therapy on bone health may be underestimated when clinical assessments rely solely on measures of bone size and density (i.e., DXA) because recovered trabecular microarchitecture may go undetected.

## Figures and Tables

**Table:** (a)

Group	Vertebra body height (mm)	Vertebra Body CSA (mm^2^)	Vertebra TMD (mgHA/ccm)
GHS	3.19 ± 0.040	1.82 ± 0.030	829.8 ± 10.1
GHD	2.28 ± 0.041**	1.17 ± 0.016**	819.5 ± 9.0
Early	2.72 ± 0.026^∗∗++^	1.39 ± 0.021^∗∗++^	840.0 ± 3.0
Late	2.69 ± 0.042^∗∗++^	1.43 ± 0.043^∗∗++^	821.0 ± 6.9
Saline	2.26 ± 0.063	1.18 ± 0.036	832.7 ± 5.8
CV%	4.9%	6.2%	2.6%

**Table:** (b)

Group	Femur length (mm)	Femur CSA (mm^2^)	Medullary CSA (mm^2^)	Femur Ct.Th (mm)	Femur TMD (mgHA/ccm)
GHS	14.97 ± 0.10	2.092 ± 0.063	1.111 ± 0.035	0.224 ± 0.005	897.9 ± 16.8
GHD	11.29 ± 0.07**	1.190 ± 0.020**	0.679 ± 0.015**	0.150 ± 0.002**	888.3 ± 12.8
Early	12.72 ± 0.08^∗∗++^	1.421 ± 0.030^∗∗+^	0.752 ± 0.017**	0.184 ± 0.004^∗∗++^	909.9 ± 5.6
Late	12.37 ± 0.08^∗∗++#^	1.429 ± 0.046^∗∗+^	0.786 ± 0.024^∗∗+^	0.176 ± 0.005^∗∗++^	905.9 ± 6.3
Saline	11.32 ± 0.04	1.218 ± 0.039	0.702 ± 0.029	0.150 ± 0.004	898.8 ± 5.9
CV%	1.8%	7.9%	8.8%	6.4%	3.3%

**Table 2:** Vertebral trabecular microarchitectural parameters including bone volume ratio (BV/TV), connectivity density (Conn.D), structure model index (SMI), trabecular number (Tb.N), trabecular separation (Tb.Sp), and trabecular thickness (Tb.Th) in GH-sufficient (GHS), GH deficient (GHD), early treatment (early; daily GH injections from age 21 to 60 days), late treatment (late; daily GH injections from age 35 to 60 days), and saline control (saline; daily saline injections from age 21 to 60 days). Mean and standard errors are reported, and the coefficient of varation (CV%). *P ≤ 0.05, **P ≤ 0.001 versus GHS, ^+^ *P* ≤ 0.05, ^++^ *P* ≤ 0.001 versus GHD.

Group	BV/TV (%)	Conn.D (1/mm^3^)	SMI	Tb.N (1/mm)	Tb.Sp (mm)	Th.Th (mm)
GHS	25.29 ± 1.05	363.8 ± 22.3	0.79 ± 0.08	5.53 ± 0.16	0.167 ± 0.006	0.0512 ± 0.0010
GHD	20.12 ± 1.38*	358.7 ± 40.1	1.33 ± 0.11*	5.84 ± 0.21	0.160 ± 0.007	0.0416 ± 0.0009**
Early	25.14 ± 1.24^+^	358.2 ± 21.6	0.91 ± 0.10^+^	6.12 ± 0.16	0.147 ± 0.005	0.0473 ± 0.0008^∗++^
Late	22.95 ± 0.99	350.7 ± 20.5	1.09 ± 0.09	6.05 ± 0.19	0.151 ± 0.005	0.0452 ± 0.0009**
Saline	21.93 ± 1.76	308.9 ± 22.7	1.23 ± 0.11	6.10 ± 0.26	0.150 ± 0.007	0.0429 ± 0.0015
CV%	16.7%	22.1%	27.7%	9.8%	6.7%	11.7%

**Table 3:** Mechanical properties in GH-sufficient (GHS), GH-deficient (GHD), early treatment (early; daily GH injections from age 21 to 60 days), late treatment (late; daily GH injections from age 35 to 60 days), and saline control (saline; daily saline injections from age 21 to 60 days). Mean and standard errors are reported, and the coefficient of varation (CV%). *P ≤ 0.05, **P ≤ 0.001 versus GHS, ^+^ *P* ≤ 0.05, ^++^ *P* ≤ 0.001 versus GHD.

Group	Femur flexural rigidity (N · mm^2^)	Vertebra axial rigidity (N)	Vertebra trabecular apparent E (MPa)
GHS	3021.2 ± 156.6	7856.1 ± 289.2	2463.5 ± 209.8
GHD	1006.9 ± 32.3**	4321.9 ± 167.8**	1545.4 ± 185.3*
Early	1432.5 ± 67.4^∗∗+^	5757.7 ± 183.9^∗∗++^	2409.0 ± 210.5^+^
Late	1439.1 ± 105.5^∗∗+^	5373.7 ± 239.2^∗∗+^	1889.6 ± 116.3
Saline	1037.2 ± 67.4	4348.8 ± 208.0	1669.2 ± 286.1
CV%	16.1%	12.0%	30.8%
